# How information processing and risk/benefit perception affect COVID-19 vaccination intention of users in online health communities

**DOI:** 10.3389/fpubh.2023.1043485

**Published:** 2023-02-21

**Authors:** Hao Liu, Liyue Gong, Cao Wang, Yunyun Gao, Yi Guo, Minhan Yi, Hao Jiang, Xusheng Wu, Dehua Hu

**Affiliations:** ^1^Department of Biomedical Informatics, School of Life Science, Central South University, Changsha, China; ^2^Shenzhen Health Development Research and Data Management Center, Shenzhen, China

**Keywords:** COVID-19 vaccine, online health community, information processing, risk perception, benefit perception

## Abstract

**Objective:**

To investigate the relationship among information processing, risk/benefit perception and the COVID-19 vaccination intention of OHCs users with the heuristic-systematic model (HSM).

**Methods:**

This study conducted a cross-sectional questionnaire *via* an online survey among Chinese adults. A structural equation model (SEM) was used to examine the research hypotheses.

**Results:**

Systematic information processing positively influenced benefit perception, and heuristic information processing positively influenced risk perception. Benefit perception had a significant positive effect on users' vaccination intention. Risk perception had a negative impact on vaccination intention. Findings revealed that differences in information processing methods affect users' perceptions of risk and benefit, which decide their vaccination intention.

**Conclusion:**

Online health communities can provide more systematic cues and users should process information systematically to increase their perceived benefits, consequently increase their willingness to receive COVID-19 vaccine.

## 1. Introduction

The COVID-19 pandemic has caused a major health crisis in humans (1). As of 13 Sep 2022, the World Health Organization has reported more than 600 million cumulative confirmed cases of COVID-19 and more than 6 million cumulative deaths (2). Ensuring that all people are vaccinated will help control the spread of COVID-19 (3) and thus protect the public from COVID-19 (4).

In China, the government legislated an emergency authorization of COVID-19 vaccine for people at high risk in June 2020 (5), and subsequently approved COVID-19 vaccines for public use in December 2020 (5, 6). As of March 2020, China has passed through its peak of the pandemic. However, China is still experiencing a small increase in cases due to the impact of COVID-19 mutations and the importation of cases from abroad. Vaccination and testing are the main vaccination policies in China. As of July 22, 2022, the first full-round vaccination rate reached 89.7% and the booster vaccination rate was 71.7% (7). Although overall the vaccination rate is relatively high. However, the booster vaccination rate is much lower compared to the first full vaccination rate. In order to strengthen the protective efficacy of the vaccine against COVID-19 mutations, ensuring the booster vaccination rate is an effective measure. In this context, continued attention to the factors influencing the intention to vaccinate against COVID-19 can inform the maintenance of the intention to vaccinate against COVID-19, the improvement of booster vaccination rates, and the development of vaccination policies.

Previous studies have explored the effect of perceived risk/benefit on intention to vaccinate for COVID-19 (8–10). For example, a study by Liora Shmueli showed that perceived benefit was the most important predictor of acceptance of the COVID-19 vaccine (11). Another study showed a strong correlation between risk perception and vaccine acceptance (12). And vaccine-related information affects users' perceptions of the risk and benefit of vaccines. Users' processing of vaccine-related information shapes their perception of vaccines (13, 14). Some studies have found that having the correct knowledge is directly related to a higher perception of risk in the older population (15). Knowledge about vaccines was associated with how individuals perceived the relevant risks and benefits of those aspects of the vaccine (16). Information related to the efficacy and safety of vaccines critically influences the acceptance of COVID-19 vaccines (4). Knowledge formation comes from information processing. However, there are no studies that have explored the effects of perceived risks/benefits on the willingness to vaccinate for COVID-19 from an information processing perspective. Processing of vaccine-related information is a key factor in the formation of people's perceived attitudes. As such, further research is necessary to determine how information processing affects risk/benefit perception associated with COVID-19 vaccination intention.

As access to information through the Internet has the advantage of being quick and convenient, online health communities (OHCs) have become one of the most important channels through which people obtain information during a pandemic (17–19). People used OHCs to learn about COVID-19 and seek information about available vaccines (20, 21). OHCs are online interactive platforms with health-related features, such as online consultation, health information exchange and experience sharing, which provide users with information and emotion support (19). Users can also benefit from OHCs by adopting healthier behaviors (22).

Therefore, in the context of the rapid development of Internet medicine and the normalization of COVID-19 prevention and control. Our study explores the impact of users' vaccine-related information processing in OHCs and their risk/benefit perception of vaccines, consequent on COVID-19 vaccination intention. Our study findings may help relevant health authorities to take more effective measures to increase vaccination rates, maintain COVID-19 vaccination intentions among Chinese residents, and provide a reference for the development of Internet healthcare.

## 2. Theoretical foundation and research hypotheses

### 2.1. Theoretical foundation

#### 2.1.1. The information behavior model

Wilson's information behavior theory suggests that users engage in information seeking through formal or informal means in order to satisfy their information needs, and then process and use the information (23). This process is influenced by activating mechanisms (e.g., stress/coping theory, risk/reward theory) and intervening variables (e.g., psychological, demographic, role-related or interpersonal). This model has been widely used in studies related to user information behavior (24, 25). Our study examines the factors associated with information behavior and the effect of perceived risk/benefit on willingness to vaccinate.

#### 2.1.2. The heuristic-systematic model

The heuristic-systematic model (HSM) of information processing includes two types of information processing: heuristic information processing and systematic information processing (26), where systematic information processing involves a more comprehensive analysis and understanding of information. On the other hand, heuristic information processing requires only simple decision rules such as intuition and experience to form judgments (27). The HSM has been widely used to explain people's attitude or behavior responses to information. The model considers information processing as a precursor to attitude formation or change, and therefore proposes two basic information processing patterns that people may adopt after acquiring information and assessing and judging risks or things.

### 2.2. Research hypotheses

#### 2.2.1. The antecedents of information processing

Information needs are also known as information insufficiency, where people lack sufficient information to make informed decisions (28). Information needs arise when the information that people want to know is more than the knowledge they have. During the COVID-19 pandemic, people need sufficient information to make decisions about whether to receive the COVID-19 vaccine (29). Previous research found that users' demand for information on the prevention of COVID-19 accounted for 36.11% in OHCs (21), which shows their great concerns and information needs about COVID-19 prevention. Some studies have suggested that information needs predict information seeking (8, 30). We assume that information needs about the COVID-19 vaccine positively influence information seeking (H1a). People satisfy their information needs by seeking information (31). In addition, if people do not have enough information to cope with emergencies, the more intense their information needs are, and the more actively they will use systematic processing (8, 32). Conversely, the heuristic processing will become more active (13, 33). Therefore, it is assumed that information needs positively influence systematic information processing (H1b) and negatively influence heuristic information processing (H1c):

Hypothesis 1a (H1a). Information needs positively influence information seeking.

Hypothesis 1b (H1b). Information needs negatively influence heuristic information processing.

Hypothesis 1c (H1c). Information needs positively influence systematic information processing.

Information seeking is a dynamic process of acquiring information and knowledge (34). People seek information through various approaches to obtain reliable information (35–37). OHCs provide a platform for people to seek and obtain information. Kahlor thinks information seeking is the precondition for information processing (38). The research of Guo found that information seeking positively affects systematic information processing (26). Information seeking intention is positively correlated with systematic processing and heuristic processing (39). We proposed that when people actively seek information about COVID-19 vaccination, both heuristic information processing (H2a) and systematic information processing will improve (H2b):

Hypothesis 2a (H2a). Information seeking positively affects heuristic information processing.

Hypothesis 2b (H2b). Information seeking positively affects systematic information processing.

#### 2.2.2. Heuristic-systematic information processing

The heuristic-systematic information processing model states that people use one or two types of information processing to help them evaluate information to make decisions (13). Most people will only make decisions based on superficial information cues (40). Systematic information processing requires more comprehensive cognition and analysis by individuals (13, 41), and the process of systematic information processing consumes more time and effort on the part of the individual. Therefore, when people carry out systematic information processing, more reliable and effective information can be obtained (26). Trumbo demonstrated that heuristic information processing negatively affects risk perception, while systematic information processing positively affects risk perception in his study about cancer (13). Smerecnik et al. (42) used an adapted HSM scale to test the relationship between information processing and risk perception about hypertension. In a study about the risk associated with the companies of a petrochemical complex, systematic processing has a direct, positive, and significant influence on risk perception (43). For benefit perception, both heuristic and systematic information processing are linked to higher benefits of using of nanotechnology (44). However, few researchers have focused on the relationships between information processing and risk/benefit perception in the context of online health communities. Therefore, we established the following hypotheses:

Hypothesis 3a (H3a). Heuristic information processing has a negative effect on risk perception.

Hypothesis 3b (H3b). Heuristic information processing has a positively impact on benefit perception.

Hypothesis 4a (H4a). Systematic information processing positively affects risk perception.

Hypothesis 4b (H4b). Systematic information processing positively affects benefit perception.

#### 2.2.3. Risk/benefit perception

With the experiment and implementation of the COVID-19 vaccine, the side effects and adverse effects of the vaccination began to appear (45), which increased people's risk perception of the COVID-19 vaccine. Kelly defined risk perception as potential adverse events or side effects from taking the drug (46). For example, myocarditis/pericarditis was a rare complication of COVID-19 mRNA vaccinations, especially in young and adolescent males (47). The lack of effectiveness of COVID-19 vaccines may also threaten people's life, thus people perceive the risk of vaccination, which will lead to vaccine hesitancy and anti-vaccination movements (48). Some scholars have found that risk perception negatively affects behavioral intentions (8, 49). This means that when people are aware of the potential risks of vaccination, they may refuse to receive it. Therefore, we proposed hypothesis H5.

Contrary to risk perception, benefit perception is considered as the perception of the benefits of vaccination, such as disease prevention and self-protection (10, 50). Wong et al. (9) and Yu et al. (10) proved the positive impact of benefit perception on vaccination intention with the health belief model. However, it has not been studied in the context of online health communities. We believed that the perceived benefit of COVID-19 vaccination information will positively affect users' willingness to vaccinate (H6):

Hypothesis 5 (H5). Risk perception will weaken OHCs users' intention to receive the COVID-19 vaccine.

Hypothesis 6 (H6). Benefit perception will increase OHCs users' intention to receive the COVID-19 vaccine.

#### 2.2.4. Model building

**Figure 1:**
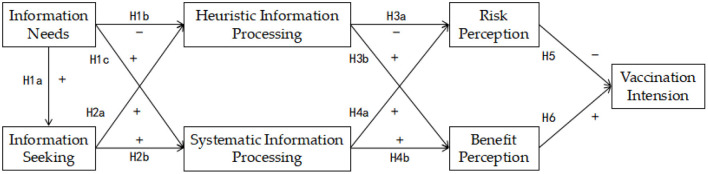
The conceptual research model. Hypothesis: H1–H6. “+” means positive effect, “–” means negative effect. The arrows point to the affected variables.

In accordance with our research hypotheses above, a new model was constructed by integrating HSM with risk/benefit perception to examine the mechanisms influencing users' willingness to vaccinate against COVID-19 in OHCs. As shown in Figure 1, in which information needs and information seeking are antecedents of information processing, information processing is assumed to predict risk/benefit perception, and risk/benefit perception directly influences vaccination intention.

## 3. Materials and methods

### 3.1. Study design

This study aims to investigate the relationship between users' information needs, information seeking, heuristic-systematic information processing, risk perception, benefit perception, and vaccination intention against COVID-19 in OHCs. We conducted an online survey *via* Questionnaire Star (https://www.wjx.cn/ accessed on 30 June 2021). The questionnaire includes two parts: the first part is sociodemographic characteristics, namely, gender, age, education level, occupation, income, and health status; the second part is the scale measurement part, using a five-point Likert scale from “strongly disagree (1)” to “strongly agree (5),” which was adapted from previous studies. We revised it into Chinese scale and pre-tested. According to the advice of the pre-test participants and the experts group, we revised some sentences and words, for example, we changed “never” into “rarely” in the heuristic information processing items. The final scale settings are shown in Table 1.

**Table 1:** Constructs and measurement items.

**Constructs**	**Items**	**Contents**	**Source**
Information needs (IN)	IN1	I need more knowledge about COVID-19 vaccination.	Ter Huurne and Gutteling (35)
	IN2	I need a lot of information to decide whether to get the COVID-19 vaccine.	
	IN3	I want to get more information about the COVID-19 vaccine from the OHCs.	
Information seeking (IS)	IS1	I am familiar with OHCs related to the COVID-19 vaccine at home and abroad.	Ter Huurne and Gutteling (35), Che and Hu (36)
	IS2	I will search for information on COVID-19 vaccinations through the OHCs.	
	IS3	I will use various methods to search for more information on the COVID-19 vaccine.	
	IS4	I will follow the latest information on COVID-19 vaccinations in the OHCs every day.	
Heuristic information processing (HIP)	HIP1	I rarely find useful information about COVID-19 vaccinations.	Smerecnik et al. (42)
	HIP2	I rarely comment on the quality of the information about COVID-19 vaccinations.	
	HIP3	I rarely consider other relevant information.	
Systematic information processing (SIP)	SIP1	I will think and follow up based on the information I get.	Smerecnik et al. (42)
	SIP2	I will think about the importance of the information about the COVID-19 vaccine.	
	SIP3	I will link COVID-19 vaccine information in the OHCs to the current COVID-19 outbreak.	
Risk perception (RP)	RP1	I think the COVID-19 vaccine will bring adverse reactions.	Costa-Font and Gil (51)
	RP2	I think the COVID-19 vaccine will bring unknown sequelae.	
	RP3	I think getting the COVID-19 vaccine is life-threatening.	
Benefit perception (BP)	BP1	I think getting vaccinated against COVID-19 will make me feel safer.	Costa-Font and Gil (51)
	BP2	I think getting the COVID-19 vaccine will reduce the spread of COVID-19.	
	BP3	I feel COVID-19 vaccination provides me with a new option for COVID-19 protection.	
Vaccination intention (VI)	VI1	I would like to get the COVID-19 vaccine immediately.	Cheng et al. (52)
	VI2	I want to get the COVID-19 vaccine in recent time.	
	VI3	I plan to get the COVID-19 vaccine shortly.	

### 3.2. Procedures and participants

Before the formal survey, this study obtained ethical approval from the Institutional Review Board of the College of Life Sciences at Central South University (Reference No. 2021-1-23). We explained the purpose and significance of our research, and provided privacy protection to all participants. All participants agreed to join this study. The investigation was administered from 1 May 2021 to 15 June 2021.

A total of 525 participants completed the questionnaire. Then we excluded the respondents who had never used OHCs before by using the option “Never used an OHC.” By removing duplicates and anomalies to ensure the validity of the data, we ended up with 410 valid questionnaires. According to the minimum sample size requirement, it must be at least 10–15 times the number of scale items (53), so our effective sample size was reasonable. Valid participants were older than 18. Among them, 64.1% are women, 89.7% of the participants have a bachelor's degree or above, and 78.5% of users are under 30 years old. Sociodemographic characteristics are presented in Table 2.

**Table 2:** Sociodemographic characteristics.

**Variables**	**Categories**	**Frequency (*N* = 410)**	**Percentage (%)**
Gender	Male	147	35.9
	Female	263	64.1
Age	18–29	322	78.5
	30–49	79	19.3
	≥50	9	2.2
Education Level	Senior high school or below	9	2.2
	Junior college	33	8.0
	University	263	64.2
	Master's degree or PHD	105	25.6
Occupation	Student	243	59.3
	Private organization staff	89	21.7
	Medical staff	19	4.6
	Others	59	14.4
Income (CNY)	< 5,000	272	66.3
	5,000–10,000	90	22.0
	>10,000	48	11.7
Health status	Poor	22	5.3
	General	152	37.1
	Good	236	57.6

### 3.3. Statistical analysis

We used the structural equation model (SEM) to test our theoretical model. SEM is a multivariate statistical technique for testing hypotheses about the influences of sets of variables on other variables (54). It can measure the interrelation of latent variables that are not directly observable and is widely used in social sciences. Latent variables are measured by their corresponding observation variables, namely, scale items. In this research, latent variables include information needs (IN), information seeking (IS), heuristic information processing (HIP), systematic information processing (SIP), risk perception (RP), benefit perception (BP) and vaccination intention (VI). Because there are many latent variables and their relationship is complex, SEM is selected for verification. SEM incorporates two analytical procedures (55). Firstly, we conducted a confirmatory factor analysis (CFA), which evaluates the measurement component of a theoretical model. After, we carried out a path analysis, which evaluates the relationship between latent variables.

## 4. Results

### 4.1. Measurement model testing

We used SPSS 26.0 and AMOS 23.0 of IBM company (Chicagao, America) to analyze the reliability and validity of the measurement model. The Cronbach Alpha coefficient (56) of the scale was 0.897, and the Cronbach Alpha coefficient of each latent variable was >0.7, indicating that the internal stability and consistent reliability of the questionnaire were good.

To further examine the convergent validity of the questionnaire, a confirmatory factor analysis was conducted by AMOS23.0 to obtain values of the average variance extracted (AVE) and standardized loadings of items. As shown in Table 3, all the standardized loadings of items were >0.6 (57). Values of composite reliability (CR) were between 0.796 and 0.870, which were higher than 0.70, indicating that the constructs have good convergent validity (54). The AVEs of all structures were greater than the benchmark value of 0.5, indicating that the overall model is valid (58).

**Table 3:** Confirmatory factor analysis results for measurement model.

**Variables**	**Items**	**Factor loadings**	**AVE**	**CR**	**Alpha**
IN	IN1	0.798	0.628	0.835	0.835
	IN2	0.779			
	IN3	0.801			
IS	IS1	0.751	0.756	0.840	0.840
	IS2	0.785			
	IS3	0.753			
	IS4	0.722			
HIP	HIP1	0.801	0.583	0.807	0.808
	HIP2	0.714			
	HIP3	0.773			
SIP	SIP1	0.739	0.615	0.827	0.826
	SIP2	0.803			
	SIP3	0.809			
RP	RP1	0.816	0.690	0.870	0.869
	RP2	0.859			
	RP3	0.817			
BP	BP1	0.828	0.647	0.846	0.844
	BP2	0.753			
	BP3	0.829			
VI	VI1	0.839	0.569	0.796	0.801
	VI2	0.762			
	VI3	0.649			

Then we tested the model's fit indicators by AMOS23.0 (55), which are showed as Table 4: the ratio of Chi-square to the degree of freedom (χ^2^/*df*) was 2.879, which was smaller than the desired threshold of 3.0. The values of the comparative fit index (CFI), incremental fit index (IFI), and Tucker-Lewis index (TLI) were 0.927, 0.928, and 0.911, respectively. Moreover, the root mean square error of approximation (RMSEA) value was 0.068, which was lower than 0.08. These figures reveal a good fit between the measurement model and the dataset.

**Table 4:** Goodness-of-fit results.

**Fit indicators**	**χ^2^*/df***	**CFI**	**IFI**	**TLI**	**RMSEA**
Recommended value	<3.0	>0.9	>0.9	>0.9	<0.08
Measured value	2.879	0.927	0.928	0.911	0.068

### 4.2. Structural equation model analysis

The results of CFA ensure the reliability of our following analysis. By using AMOS 23.0 to set up the structural model, a path analysis was performed to test the relationships among the constructs in the model framework. The standard path coefficients (β) and *p*-value can be seen in Table 5. All the hypothesized relationships were supported, except H3a, H3b, and H4a. Information needs had a positive effect on information seeking (H1a: β = 0.66, *p* < 0.001) and systematic information processing (H1c: β = 0.42, *p* < 0.001). Hence, H1a and H1c were supported. The relationship between information needs and heuristic information processing was the opposite (H1b: β = −0.31, *p* < 0.001), so H1b was supported. Information seeking had a positive effect on heuristic information processing (H2a: β = 0.72, *p* < 0.001) and systematic information processing (H2b: β = 0.41, *p* < 0.001). Thus, H2a and H2c were supported.

**Table 5:** The results of path coefficient.

**Hypotheses**	**Path**	**Standard path coefficients**	**Standard errors**	***p*** **-value**	**Results**
H1a	IN → IS	0.66	0.07	<0.001	Supported
H1b	IN → HIP	−0.31	0.11	<0.001	Supported
H1c	IN → SIP	0.42	0.06	<0.001	Supported
H2a	IS → HIP	0.72	0.10	<0.001	Supported
H2b	IS → SIP	0.41	0.06	<0.001	Supported
H3a	HIP → RP	0.61	0.06	<0.001	Not Supported
H3b	HIP → BP	−0.02	0.04	0.644	Not Supported
H4a	SIP → RP	−0.06	0.08	0.217	Not Supported
H4b	SIP → BP	0.73	0.07	<0.001	Supported
H5	RP → VI	−0.08	0.06	<0.05	Supported
H6	BP → VI	0.84	0.04	<0.001	Supported

**Figure 2:**
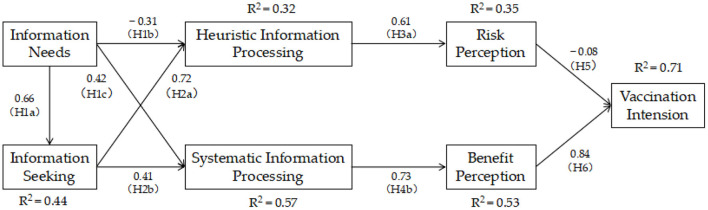
The model of final research results, including path coefficients (β), and explained variances (*R*^2^). For example, *R*^2^ = 0.44, means that the predictors of information seeking explain 44% of its variance.

The results show that heuristic information processing had a positive relationship with risk perception (H3a: β = 0.61, *p* < 0.001), since this was contrary to our hypothesis, and it had an insignificant negtive effect on the benefit perception (H3b: β = −0.02, *p* = 0.644 > 0.05). Thus, H3a and H3b were not supported. Systematic information processing had a positive effect on benefit perception (H4b: β = 0.73, *p* < 0.001), so H4b was supported. While systematic information processing to risk perception was insignificant (H4a: β = −0.06, *p* = 0.217 > 0.05), so H4a was not supported. Finally, risk perception had a negative impact on the vaccination willingness of OHCs users (H5: β = −0.08, *p* = 0.046 < 0.05), and benefit perception had a significant positive impact on the vaccination willingness of OHCs users (H6: β = 0.84, *p* < 0.001), indicating that H5 and H6 were supported. The model of final research results was as seen in Figure 2.

## 5. Discussion

### 5.1. Main findings

Our research found a strong positive correlation between vaccine information needs and information seeking among users of OHCs. The COVID-19 pandemic has severely affected the everyday life of people around the world. Even though COVID-19 sometimes mutates, vaccines are still an effective means of prevention (59), and there is often uncertainty about people's attitudes toward emerging technologies (30, 60). When it comes to the COVID-19 vaccine, this manifests itself as concerns about the safety and efficacy of the vaccine (61). To reduce uncertainty, people require accurate and effective information, which leads to further product information seeking and information processing behaviors. Savolainen (62) thinks that information needs are the fundamental factor that motivates people to identify and access information sources and the driver that stimulates them to continuously search for information. Our study adds to the empirical evidence, in which vaccine information seeking behavior was largely explained by information needs (*R*^2^ = 0.44). A study by Zhou (63) found that pandemic risk stimulates information needs and thus positively influences information seeking behavior. This point is consistent with the findings of our study.

The relationship between vaccine information needs and information processing styles was also explored in this study. The need for vaccine-related information positively influenced systematic information processing, and negatively influenced heuristic information processing. According to Griffin et al. (32), people are more likely to process information systematically when they have a greater desire for information. Hubner and Hovick (64) proved that information insufficiency is positively associated with systematic processing. Our study implies that Griffin's model also applies to users' vaccine-related information processing within the online health community. On the other hand, our work reflects that users prioritize risk information when they look for vaccine-related information. Users need comprehensive information to determine vaccination risks and to make vaccination decisions. In this process, information seeking positively correlated with both heuristic and systematic processing. This result suggests that information seeking behavior further facilitates information processing behavior, which is the same as Zhu et al. (39)'s research findings.

The focus of this study was to investigate the relationship between information processing, risk/benefit perception, and vaccination intention. Heuristic information processing negatively influences vaccination intention of OHCs users by positively affecting their risk perception, and systematic information processing positively influences vaccination intention of OHCs users by positively affecting their benefit perception. Fast, intuition-based information processing is more likely to elicit users' perception of risk. This feature may be influenced by a large amount of information on vaccine side effects available on the Internet. When people employ heuristic information processing, they verify information less from multiple sources. In addition, because heuristic information processing makes it easier to make quick decisions, it is more difficult for them to spend time searching for more comprehensive information. On online communication platforms, people tend to spread the side effects of vaccines more often than the positive effects of vaccines (65). Concerns about the side effects are a barrier to achieving high vaccination rates (66, 67), and the perceived risk of vaccines reduces the willingness of people to receive COVID-19 vaccines.

A comprehensive and systematic approach to information processing helps people to perceive the benefits of vaccines and thus promotes their intention to receive vaccinations, and Jing et al. found that parents were more likely to accept childhood vaccinations when they systematically described and processed information (68). Jing suggested that systematic information processing could lead parents to adopt an “objective” or “balanced” approach, which is compatible with their perceived benefits of childhood vaccination, thus promoting vaccination (68). Our findings further establish the relationship between systematic information processing, perceived benefits, and vaccination intentions. Another study concluded that active participation in information behavior helps reduce the public's uncertainty and mitigate risk about COVID-19 pandemic (69). In our study, users more actively involved in information dissemination were more likely to have comprehensive exposure to vaccine information, adopt systematic information processing, and enhance perceived benefits, thus increasing their vaccination intention.

Our study also found that systematic information processing failed to positively influence perceived risk. In contrast, a previous study showed that systematic processing positively influenced perceived risk and thus protective behavioral intentions (49), which is inconsistent with our findings. The possible reason for this is that the information processing in their study was for the vaccine scandal, whereas the participants in our study were exposed to comprehensive vaccine information. Participants who used more systematic processing may have been more likely to use authoritative, official information, whereas authoritative information in China showed more benefits of vaccination. The failure of heuristic processing to predict benefit perception may also be related to the rapidity of the information processing subject. The way decisions are made are based on surface information cues, and the complexity of internet information.

### 5.2. Implications

In the context of the global COVID-19 pandemic, we combined information behavior theory and the heuristic-systematic information processing model to study the COVID-19 vaccination intention of OHC users. Compared with previous studies, we innovatively explored the relationship between cognitive processing and risk/benefit perceptions from two pathways (heuristic or systematic processing). It also provides new theoretical guidance for the application of HSM. Also our study found that the online health community, an information platform, plays a significant role in disseminating information and improving users' vaccination confidence during the pandemic. It provides a basis in the literature for using the network platform to promote public health.

There are several practical applications of our research to increase people's intention to vaccinate against COVID-19. Firstly, the government administration should strengthen the regulation of information about the COVID-19 pandemic and propagandize knowledge of COVID-19 vaccine among the public. Secondly, managers of online health communities should provide users with systematic and positive clues, such as more informative and effective information (e.g., a list of vaccination places, time or price). They are also recommended to expand the retrieval systems of OHCs so that searching is as easy as possible for users. In addition, both government administration and OHCs managers should enhance the dissemination of benefit-related information. Appropriate risk information is needed but must be truthful, as it can stimulate informative behavior and thus promote vaccination intentions. Finally, users should be more proactive in adopting a systematic approach to information processing and making comprehensive judgments about information, for example, by comparing information from different sources (experts, other users or third-party organizations).

## 6. Conclusion

The study confirmed the adaptability of HSM in the background of online health communities and the COVID-19 pandemic, where information needs and information seeking remain the antecedents of information processing. Differences in how OHCs users process information cause differences in perception, with heuristic information processing leading to risk perception and systematic information processing leading to benefit perception. In contrast, risk perception and benefit perception directly influence OHCs users' willingness to vaccinate against COVID-19. Although the negative effect of risk perception on vaccination intention is small but present, the positive effect of benefit perception on vaccination intention is much more significant.

## 7. Limitations and future work

There are some limitations of this study. First, the study was only on Chinese online health community users. Therefore, our findings lack generalizability. Considering the differences between China and foreign countries in terms of COVID-19 pandemic prevention policies and internet management, the impact of online health communities in different countries can be further studied in the future. Second, our quantification of user information behavior through scales may be subjective. More objective measurement tools (i.e., eye-tracking) or methods (i.e., in-depth interview method) could be used further in future studies. Third, cross-sectional studies are limited to confirming the relationship between variables at a particular time period. As vaccination intentions of OHCs users may vary with the development of the COVID-19 pandemic and policies, longitudinal studies can be adopted in future studies to discover further changes in users' vaccination intentions against COVID-19. Finally, since our target population are people who frequently use online health communities and can agree to participate in our survey, some people will refuse to participate in the survey due to disease privacy concerns. Although our sample size meets the minimum requirements for SEM, it is still relatively small. Future studies can expand the sample size; additionally, studies might investigate populations not using online health communities to obtain more comprehensive conclusions.

## Data availability statement

The original contributions presented in the study are included in the article/supplementary material, further inquiries can be directed to the corresponding author.

## Ethics statement

The studies involving human participants were reviewed and approved by Institutional Review Board of College of Life Sciences, Central South University. The patients/participants provided their written informed consent to participate in this study. Written informed consent was obtained from the individual(s) for the publication of any potentially identifiable images or data included in this article.

## Author contributions

HL and DH: conceptualization and methodology. HL, LG, CW, and YuG: software, validation, investigation, and formal analysis. YiG and MY: resources and data curation. HL and LG: writing—original draft preparation. HJ, XW, and DH: writing—review and editing. DH: visualization, supervision, project administration, and funding acquisition. All authors have read and agreed to the published version of the manuscript.
